# Establishing DNA‐Based Strategies for Soil Biodiversity Assessment: Insights From Carabid Beetles

**DOI:** 10.1002/ece3.72461

**Published:** 2025-11-06

**Authors:** Luísa Fraga Dornellas, Vanessa A. Mata, Sara Mendes, Ricardo Leitão, Marie Bartz, Eduardo Nascimento, Joana Costa, José Paulo Sousa, Luís Cunha

**Affiliations:** ^1^ Centre for Functional Ecology, Associate Laboratory TERRA, Department of Life Sciences University of Coimbra Coimbra Portugal; ^2^ CIBIO, Centro de Investigação em Biodiversidade e Recursos Genéticos, InBIO Laboratório Associado Universidade do Porto Vairão Portugal; ^3^ BIOPOLIS Program in Genomics, Biodiversity and Land Planning CIBIO Vairão Portugal; ^4^ Centre for Organic and Regenerative Agriculture Idanha‐a‐Nova Portugal

**Keywords:** Carabidae, metabarcoding, methodological comparison, mock community, soil macrofauna, taxonomy

## Abstract

Molecular‐based methods offer valuable opportunities for assessing soil biodiversity in different ecosystems. However, their reliability and large‐scale applicability depend on developing, optimizing protocols and establishing high quality, curated local reference databases. This study aimed to evaluate key steps in soil macroinvertebrate metabarcoding workflow, including the sample decontamination process and the efficiency of taxa recovery. Specifically, we sought to: (1) determine the impact of sample decontamination, (2) validate species‐level recovery efficiency of the metabarcoding pipeline spiked with a curated mock community of morphologically identified and barcoded carabid beetles, and (3) compare traditional morphological identification and metabarcoding for specimens' taxonomic assignment and recovery. Our results showed that the commonly used decontamination process did not significantly impact OTU richness, suggesting it is not essential for this fauna. Compared to morphology, to metabarcoding provided a more comprehensive taxonomic overview at higher level taxa. However, validation with the mock community revealed discrepancies in species‐level recovery, underscoring that its accuracy is highly contingent on the quality of the reference database. DNA metabarcoding is a currently used and promising technique for macroinvertebrate assessment regarding time, efficiency, and costs, yet reaching greater depth in taxonomic resolution. Yet, its species‐level accuracy remains dependent on comprehensive and well‐curated barcode reference databases. We recommend an integrative approach, combining molecular data with targeted validation, for the most robust outcomes. For this reason, we recommend the use of integrative methodologies for robust and rapid biodiversity assessments. We found that the common decontamination step is not crucial for soil macrofauna metabarcoding accuracy. Consequently, its removal streamlines sample processing.

## Graphical abstract

DNA metabarcoding revolutionizes soil biodiversity assessments by offering unparalleled taxonomic resolution and efficiency compared to traditional methods. Our study shows that the common decontamination step is unnecessary, streamlining workflows without compromising accuracy. However, the reliability of these molecular methods hinges on the development of curated local reference databases, underscoring the importance of integrative approaches for robust biodiversity monitoring.

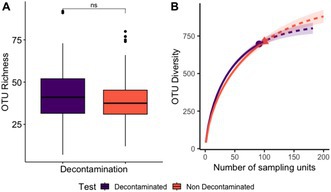

## 1 Introduction

Soil is the foundation of terrestrial ecosystems; it supports multiple functions fundamental for the balanced functioning of ecosystems and its critical role in terrestrial ecosystems is widely recognized by organizations, public entities, and governments (European Commission 2021). In Europe, 60%–70% of soils are considered unhealthy, with ongoing degradation processes causing significant damage each year (European Commission 2021). This has prompted an urgent call for soil preservation and restoration efforts, alongside the development of new policies. Moreover, the complexity and modularity of the soil system provide different ecological niches supporting an astonishing range of biodiversity (FAO 2020; Stolte et al. 2016). Indeed, soil is likely one of the most biodiverse environments across terrestrial ecosystems, holding nearly a quarter of Earth's species (Arribas et al. 2021; Decaëns et al. 2006; FAO 2020; Guerra et al. 2021), consequently being considered a global reservoir of biodiversity (Arribas et al. 2021; FAO 2020; Guerra et al. 2021).

Biodiversity loss has been widely reported across various ecosystems, and soils are not immune to this trend, with a direct impact on ecosystem services (Delgado‐Baquerizo et al. 2025; Jeffery et al. 2010; Phillips et al. 2024). Soil biodiversity can be defined as the variety of life belowground, from genes to communities; these organisms play crucial roles in maintaining and supporting soil functions and, therefore, the goods and services provided by soils (FAO 2020). Indeed, soil biodiversity loss is a threat European soils are facing (European Commission 2021). Soil biodiversity not only contributes to the health of soils, but it may also be used to improve ecosystem functionality in a way that allows more efficient use of pesticides and fertilizers, cutting down management costs (European Commission 2021). Accordingly, setting fast and reliable monitoring schemes is of utmost importance to understand the impact of changes in management practices.

Additionally, there is a clear taxonomic bias in biodiversity research. Soil biodiversity remains understudied compared to other taxa, such as aboveground organisms (Bardgett and Van Der Putten 2014; Cameron et al. 2018; Decaëns 2010; Wall 2012). Explicitly, the classes Arachnida and Insecta contain the least represented species groups in the Global Biodiversity Information Facility (GBIF) database when compared to the number of known species (Troudet et al. 2017). More broadly, research on most soil‐associated taxa remains sparse, highlighting a significant gap in our understanding of these critical species (Phillips et al. 2020; Salis et al. 2024). Despite this bias, most classes over‐ and underrepresented in 1950 remained the same in 2016 (Troudet et al. 2017), further complicating soil conservation (Cameron et al. 2018).

Monitoring soil biodiversity is therefore critical for assessing ecosystem health, guiding sustainable land management and fighting against biodiversity loss. However, traditional assessments based on morphological identification of macrofauna are time‐consuming, and expensive (FAO 2020; Gongalsky 2021; Jeffery et al. 2010), and rely on taxonomic expertise (Arribas et al. 2022; Gongalsky 2021; Watts et al. 2019), a resource limited by the well‐documented “taxonomic impediment” (Cao et al. 2016; Gongalsky 2021; Valdecasas and Camacho 2003). These methods struggle with cryptic species, immature life stages, and damaged specimens, creating a significant bottleneck that limits the scale, accuracy and frequency of biodiversity surveys. Furthermore, the lack of a global consensus on sampling and methodological approaches to assess soil biodiversity (Cameron et al. 2018; Guerra et al. 2022) delays or impairs dataset comparison (Cameron et al. 2018).

Molecular techniques, such as DNA metabarcoding, have emerged as a powerful alternative that overcomes many of the traditional methods' limitations. They have already been implemented across different ecosystems, including freshwater (Baselga et al. 2013; Bista et al. 2018), marine (Fonseca et al. 2010; Leray and Knowlton 2015), terrestrial (Arjona et al. 2022; Ji et al. 2013; Martoni et al. 2023; Mata et al. 2021; Watts et al. 2019), and paleoenvironments (Cao et al. 2020).

By combining DNA barcoding and high‐throughput DNA sequencing (HTS), DNA metabarcoding can offer rapid, consistent, and cost‐effective identification of organisms from bulk community samples. This approach significantly increases the amount of biodiversity data while reducing the reliance on manual taxonomic sorting for broad‐scale assessments (Gongalsky 2021; Ji et al. 2013). Arthropod wocDNA metabarcoding is currently widely used to assess arthropod communities (Arribas et al. 2021; Creedy et al. 2022). Despite its potential, harmonized and established protocols are still lacking, meaning that results can vary significantly based on methodological choices (Creedy et al. 2022). Sample collection, handling, and storage processes for molecular‐based approaches are fundamental and must be carefully considered to avoid DNA contamination and ensure DNA preservation (Arribas et al. 2022; Liu et al. 2020). Moreover, sample decontamination is a common step before DNA extraction in metabarcoding protocols. It is used to minimize contamination between samples by removing exogenous DNA that might be present in bulk samples, increasing methodological reliability (Hausmann et al. 2021; Jüds et al. 2023). Despite its importance, sample decontamination is time‐consuming and can potentially damage the targeted DNA content. Additionally, DNA metabarcoding reliability depends on the existence of curated reference databases.

This study aims to evaluate and refine the DNA metabarcoding workflow for soil macroinvertebrate assessment from Central Portugal and enhance biodiversity monitoring and conservation efforts by leveraging the strengths of both molecular techniques and traditional taxonomic expertise. Our specific objectives were to:

1. Evaluate the cost–benefit relationship of hypochlorite decontamination and assess its impact on contamination levels.
2. Validate species recovery efficiency of the metabarcoding pipeline using a curated mock community of morphologically identified and barcoded carabid beetles.
3. Compare the taxonomic diversity and resolution achieved by DNA metabarcoding against results from traditional morphological identification.

## 2 Materials and Methods

### 2.1 Sample Collection and Processing

This study was conducted in Idanha‐a‐Nova, Portugal, as part of the CULTIVAR project. The sampling design consisted of 24 monitoring plots, each containing nine pitfall traps arranged in a 3 × 3 grid. This layout resulted in a total of 216 pitfall traps across all plots. Pitfall traps were deployed at the end of the fall season, from late November to early December 2022, and remained in the field for 13–17 days. Pitfall traps were filled with 0.3 L of ethylene glycol, and lids were used to minimize rainwater accumulation and preservative dilution and to maintain specimen integrity. After collection, the samples were stored in 96% ethanol at room temperature until further analysis. Samples were sorted under a low‐power stereomicroscope, where specimens were counted and morphologically identified to the lowest feasible taxonomic level (typically Order), based on available taxonomic expertise and standard literature (Quigley and Madge 1988). Higher level taxonomic assignments were made using shared diagnostic traits and dichotomous keys. All identifications were verified by an expert taxonomist (coauthor Sara Mendes), and in cases of uncertainty, additional consultation was sought from specialists within our collaboration network. In several cases, specimens were retained at higher taxonomic ranks (e.g., family or order) due to limited morphological resolution or lack of sufficient expertise for finer identification.

### 2.2 Establishment of a Local COI Barcode Reference Database for Carabid Species Identification

Specimens belonging to the Carabidae family were identified at the species level based on morphological traits (Aguiar and Serrano 2012). To prevent cross‐contamination among samples during specimen handling, all tools, including tweezers, were thoroughly cleaned and flame‐sterilized before each use. A local barcode reference database for the cytochrome oxidase subunit 1 (COI) was generated for at least one individual representative of each identified Carabid species using the 710 bp COI sequence amplified by the Folmer primers, LCO1490: 5′‐GGTCAACAAATCATAAAGATATTGG‐3′ and HC02198: 5′‐TAAACTTCAGGGTGACCAAAAAATCA‐3′ (Folmer et al. 1994). For this, individual specimens were removed from 96% ethanol, let dry for 10 min on tissue paper, and then bleached with a 1% sodium hypochlorite (NaOCl) solution by gently shaking the specimen in a 2 mL tube for 3 min (Palmer‐Young et al. 2019). Subsequently, the bleach was discarded, and individuals washed in distilled water three times for 1 min each (Hausmann et al. 2021). DNA extraction was done using a Qiagen DNeasy Blood & Tissue Kit (QIAGEN, Hilden, Germany) following the manufacturer's instructions, with some adaptations. The DNA extraction protocol varied according to specimen size. For small individuals (< 1 cm), a nondestructive approach was used, where the entire organism was immersed in a digestion buffer overnight. For larger specimens (> 1 cm), DNA was extracted destructively using 1–3 appendices from a single side of the specimen, which were removed and macerated. DNA quality check was done using a Nanodrop and quantified using Qubit (Thermo Fisher Scientific, Waltham, MA, USA).

For the PCR, 10 μL of Supreme NZYTaq II 2× Green Master Mix (NZYtech, Portugal), 1 μL of each primer (10 nM) and at least 10 ng/μL DNA template were used in each reaction in a total volume of 20 μL per reaction. Cycling conditions consisted of an initial denaturation at 95°C for 3 min and five cycles of denaturation at 95°C for 30 s, annealing at 46°C for 30 s, and extension at 72°C for 45 s, followed by 32 cycles of denaturation at 95°C for 30 s, annealing at 51°C for 30 s, and extension at 72°C with a final elongation at 72°C for 10 min. The PCR products were checked on a 1% agarose gel to verify the amplification success and purified with ExoSAP‐IT Express (Applied Biosystems by Thermo Fisher Scientific). Sanger sequencing of the purified PCR products was performed (Eurofins, Germany). COI barcode sequences were used as a query in a search against the BOLD database (Barcode of Life Data System: https://boldsystems.org/), using the default query coverage of ≥ 98%, and taxonomically assigned to the corresponding species identification where possible. Sequences lacking a taxonomic assignment on BOLD were further analyzed through the BLASTn algorithm (Altschul et al. 1990; Camacho et al. 2009) from the GenBank database (Sayers et al. 2022), with a minimum query coverage threshold of 97%.

### 2.3 Bulk Sample Preparation, DNA Extraction, and Library Preparation for COI Metabarcoding

Following the morphological sorting, all the identified macrofauna species, except for individuals from the Carabidae and Formicidae families, were pooled back together by sample. This resulted in 191 bulk community samples. When handling specimens, all materials, such as tweezers and spatulas, were cleaned with sodium hypochlorite (3%) and 96% ethanol. Before DNA extraction, half of the bulk samples were decontaminated with a 3% sodium hypochlorite solution for 1 min to reduce exogenous DNA and were washed three times with distilled water for 1 min (Hausmann et al. 2021). Bulk samples were then air‐dried overnight in an incubator at 56°C. According to the amount of biological content, one to four glass beads of 8 mm diameter were added to each bulk sample to turn the biological material into a fine powder through a homogenizing process using the Bullet Blender 50‐DX homogenizer (Next Advance, NY, USA) for at least 15 min. DNA extraction was performed using the E.Z.N.A. Tissue DNA Kit (Omega Bio‐Tek, GE, USA), following an adapted protocol (Mata et al. 2021). For each sample, DNA was extracted from 70 mg of homogenized bulk insect powder. If < 70 mg of sample was available, the entire sample was used. We excluded two taxa from the metabarcoding workflow: ants (Formicidae) and carabid beetles (Carabidae). Ants were set aside for a different project, and the carabid beetles were used to make a mock sample to validate the metabarcoding pipeline. A two‐step PCR protocol was followed for Illumina library preparation. For the first PCR, BF3 (CCHGAYATRGCHTTYCCHCG) and BR2 (TCDGGRTGNCCRAARAAYCA) primer pair with Illumina adapters was used, which amplifies the 418 bp amplicon fragment of the cytochrome oxidase I (COI) mitochondrial gene (Elbrecht et al. 2019). This PCR comprised 5 μL of Qiagen Multiplex Master Mix (QIAGEN, Hilden, Germany), 0.3 μL of each 10 nM primer (BF3‐BR2), 3.4 μL of H_2_O, and 1 μL of template DNA, previously diluted 1:100. Cycling conditions consisted of initial denaturation at 95°C for 15 min, followed by 35 cycles of denaturation at 95°C for 30 s, annealing at 45°C for 30 s, and extension at 72°C for 30 s, with final elongation at 60°C for 10 min. PCR products were checked on 2% agarose gel to verify the amplification success. Throughout the laboratory workflow, extraction blanks and PCR negative controls were included to assess and control for potential contamination. All samples produced visible amplification bands, while extraction blanks and PCR negative controls showed no signs of contamination. PCR products were diluted 1:4 and a second PCR was performed to incorporate the 7‐bp‐long identification indexes based on Illumina Nextera XT Kit (Mata et al. 2019), and Illumina P5 and P7 sequencing adapters. PCR conditions were similar to the first PCR, except that 7 μL of Kapa HiFi Hot Start mix (Roche, Basel, Switzerland) was used, as well as 0.7 μL of each 10 nM indexing primer. Cycling conditions consisted of initial denaturation at 95°C for 3 min, followed by eight cycles of denaturation at 95°C for 30 s, annealing at 55°C for 30 s, and extension at 72°C for 30 s, with a final elongation at 72°C for 5 min. Following the second PCR, the resulting amplicons for each sample were purified on the left side using Agencourt AMPure XP beads (Beckman Coulter, Brea, CA, USA). The concentration of each purified sample was then quantified using Nanodrop. To achieve normalization, each sample was diluted to a standard concentration of 20 nM. These normalized PCR products were then pooled into a single equimolar library. Finally, the concentration of this library was quantified using qPCR with KAPA Library Quant Kit qPCR Mix (Roche). The final library was sequenced on an Illumina Novaseq Platform at Novogene (Cambridge, UK). The dual PCR steps and conditions are illustrated in Appendix S1: Figure S1.

### 2.4 Mock Community Assembly and Evaluation of OTU Recovery Efficiency

Extracted DNA from each representative carabid species, 31 species, was pooled in equimolar proportions after measuring with a Qubit fluorometer (Thermo Fisher Scientific). This mock community sample served as a control to validate the metabarcoding workflow, particularly in assessing primer performance, taxonomic resolution, and estimation of OTU richness. This community was designed to evaluate the efficiency of metabarcoding pipelines in the recovery efficiency of known taxa sequences by comparing the expected species diversity with the observed OTU richness; we assessed the accuracy of taxonomic recovery within this pipeline.

To assess the impact of sample diversity complexity, we selected 30 DNA extracts of bulk community samples from both high‐diversity (*n* = 15) and low‐diversity community samples (*n* = 15). For each of these 30 base samples, an aliquot was taken and spiked with DNA from the carabid mock community sample in varying proportions, 10%, 25%, and 50%, with five samples for each combination. The original, unspiked base DNA sample was processed alongside its spiked counterpart and remaining DNA bulk community samples, serving as the 0% mock community control. This paired design allowed for a direct comparison of the community profile before and after the addition of the mock community DNA.

### 2.5 Bioinformatic Pipeline

The following bioinformatic pipeline is schematized in Appendix S1: Figure S2, upon receipt of the raw sequencing data, we conducted an initial quality assessment using FastQC (version 0.12.1; available at https://github.com/s‐andrews/FastQC) to evaluate read quality, adapter content, and other basic metrics prior to downstream bioinformatic processing. OBITools 4 (Boyer et al. 2016) was used for the sequence processing, coupled with VSEARCH (Rognes et al. 2016, 20) and LULU (Frøslev et al. 2017) for denoising. First, paired‐end reads were merged using “obipairing,” sequences that failed to align were discarded. Reads were assigned to their respective samples and primer sequences were trimmed using the “obimultiplex” command, allowing for up to four mismatches per primer sequence. Reads were then dereplicated into unique sequences with “obiuniq,” and singletons (sequences with only one read per sample) were removed using “obigrep.”

Then, the remaining exact sequence variants (ESVs) were combined into a single file and processed with VSEARCH. First, reads were dereplicated again using the command “‐‐derep_fulllength.” Then denoised with “‐‐cluster_unoise,” where reads were removed attributed to PCR and sequencing errors. Subsequently, chimeric sequences were detected and removed with “‐‐uchime3_denovo.” The remaining sequences were then grouped by a 99% similarity criterion using “‐‐cluster_size,” defining the operational taxonomic units (OTUs). Putative NUMTs (nuclear copies of the mitochondrial COI) and other artifacts were then identified and curated using LULU (Frøslev et al. 2017). This curation is a fundamental step to avoid artificially inflating diversity estimates with pseudogenes or PCR artifacts. This curation reduced the OTU count by effectively removing spurious variants. All remaining OTUs were taxonomically assigned by comparing the representative OTUs of each cluster against online databases (BOLD and NCBI) through “boldigger‐cline ie_coi” command (Buchner and Leese 2020).

### 2.6 Comparative Analysis of Taxonomic Identification Methods and Statistical Validation in Soil Macrofauna Metabarcoding

Although some groups were identified with lower taxonomic levels through morphology, all statistical analyses were conducted at the lowest feasible taxonomic level, mostly at the Order level. Specimens that could not be reliably identified through morphological characteristics, including larvae, pupae, or damaged individuals, were excluded from the morphological dataset. For the metabarcoding data further filtering of sequences was needed after taxonomic assignment. OTUs assigned to taxa outside the scope of soil macrofauna or assigned only at a rank higher than Class were excluded. Additionally, we retained only those OTUs that showed a percent sequence identity of > 85% to their best match in the BOLD reference database.

Data resulting from DNA extraction blanks and PCR negatives were assessed for contaminations (Appendix S1: Figure S3). After quality filtering, OTUs with > 20 reads in any negative control were flagged and removed from the final dataset, ensuring the robustness of the resulting taxonomic assignments. This conservative threshold helped minimize the inclusion of spurious sequences and contributed to the reliability of downstream analyses.

A two‐sample *t*‐test was conducted to evaluate the comparability of sample richness between samples decontaminated with NaOCl solution and non‐decontaminated samples.

To assess the overall adequacy of the sequencing depth for each sample, rarefaction curves were generated on the number of sequence reads per sample using the “rarecurve” function (Appendix S1: Figure S4). Additionally, to evaluate the overall completeness of our methodology, we estimated the total taxonomic richness for our dataset using nonparametric species richness estimators. We used the “specpool” function in our final, filtered OTU community matrix (191 samples × 857 OTUs) to calculate the observed richness and the estimated total richness using the Chao2 estimator. The percentage of community recovery was then calculated as: (Observed Richness/Chao2 Estimated Richness) × 100.

To evaluate the informative value of occurrence and abundance data derived from both morphological and metabarcoding approaches, we visualized the data at the individual sample and population levels using biodiversity metrics adapted from Deagle et al. (2019). To ensure comparability between the two methods, we harmonized the taxonomic groupings across both datasets. Additionally, individuals from the orders Coleoptera and Hymenoptera were excluded from the morphological (order‐level abundance) and metabarcoding (order‐level read abundance) datasets. This step was essential to reduce potential bias and enable a more balanced comparison of the methods. At the sample level, we analyzed 20 randomly selected samples to assess three key metrics: order richness (i.e., the number of taxonomic orders detected per sample), weighted occurrence, and relative taxa abundance (RTA) based on morphological counts, alongside relative read abundance (RRA) derived from metabarcoding data. At the community level, we calculated frequency of occurrence (FOO), defined as the proportion of samples in which each taxon was detected; percent of occurrence (POO), a rescaled presence/absence metric; and weighted percent of occurrence (wPOO), which accounts for variation in richness by assigning equal weight to each sample. These metrics allowed us to systematically compare how each method reflects both the presence and relative abundance of taxa across samples and at broader ecological scales. In addition, we conducted a Pearson correlation analysis at the Order level. For each taxonomic Order (Coleoptera and Hymenoptera included), the morphological abundance (total number of individuals per sample) and the molecular abundance (total number of sequence reads from all OTUs assigned to that Order per sample) were compared. To normalize the data, both abundance counts and read counts were log_10_‐transformed prior to analysis. The relationship between these two metrics was then evaluated for each Order using Pearson's correlation coefficient (Appendix S1: Figure S5).

Carabid species‐level taxonomic assignments were used for methodological comparisons between traditional, barcoding, and metabarcoding methods. To define an appropriate similarity threshold for species‐level assignments within Carabidae, we aligned a curated dataset of 671 COI sequences obtained from NCBI (Appendix S2) using ClustalW (Thompson et al. 1994) in MEGA version 11.0.10 (Tamura et al. 2021). We then applied the ASAP (Assemble Species by Automatic Partitioning) method (Puillandre et al. 2021) using the Kimura 2‐parameter model (Kimura 1980; see Appendix S1: Figure S6). The optimal partitions identified by ASAP corresponded to pairwise distance thresholds ranging from 0.031 to 0.078. Based on one of the best‐supported partitions, we selected a conservative threshold of 93% similarity (i.e., 0.071 distance) for tentative species‐level identifications. This threshold was chosen to reflect the relatively short fragment length and variability within our reference set. Six sequences falling below this threshold were excluded from species‐level assignments.

For the COI barcode reference database for carabid species, we used the criteria developed by Conrado et al. (2023), to delimit the OTUs and species (Appendix S1: Table S2). Here, species where both morphological and molecular data were available were defined as Integrated Operational Taxonomic Units (IOTUs) and species identification that relied only on molecular data was separated as Molecular Operational Taxonomic Units (MOTUs). This classification allowed the creation of a Venn diagram for comparing the three methods used.

All statistical analyses and data visualization were performed in R Version 2023.06.0 + 421 using packages “iNEXT” v3.0.0 (Hsieh et al. 2016) for diversity estimates, ggplot2, v3.4.2 (Wickham et al. 2024), ggpubr v0.6.0 (Kassambara 2023), vegan v2.6‐10 (Oksanen et al. 2015) for rarefaction curves and estimation of the percentage of community recovery. For data visualization, ggVennDiagram v1.12 (Gao et al. 2021, 2024) was used for the creation of a Venn diagram. Data, code, and bioinformatic pipelines used in this study are available on Dryad through the link https://doi.org/10.5061/dryad.g1jwstr28.

## 3 Results

### 3.1 Bioinformatics Quality Measures

The Illumina sequencing run yielded a total of 34,469,580 raw paired‐end reads. Bioinformatic processing, which included quality filtering, denoising, chimera removal, and LULU curation, produced a final dataset of 19,200,919 high‐quality reads clustered into 2137 curated OTUs (Appendix S1: Table S1). A final taxonomic filtering step was applied to retain only macrofauna OTUs with a percentage of sequence identity of > 85% against the reference sequences in the BOLD database, retaining 868 OTUs and 16,052,242 reads. Reads with a high abundance in control samples > 20 reads were filtered out as contaminants (Mata et al. 2021). This resulted in a final dataset of 11,952,301 reads across 861 OTUs for downstream analysis with a mean of ~46,000 reads per sample OTUs (Appendix S1: Table S1). The total amount of reads, and read loss through the bioinformatic pipeline and associated filtering steps, as well as the identified and curated OTU steps, can be consulted in Appendix S1: Table S1.

The sequencing depth was found to be sufficient for capturing the taxonomic diversity within individual samples, as indicated by the asymptotic shape of the sample‐based rarefaction curves (Appendix S1: Figure S4). From a total of 861 OTUs in our final dataset, 857 were observed at least once across the 191 samples (excluding: controls, samples with added mock community and the mock sample). Based on the Chao2 richness estimator, this observed richness represents an estimated 89.7% of the total projected richness for the sampled habitats (estimated total richness = 955.7 ± 19.3 SE).

### 3.2 Sample Collection and Specimens Identification

**FIGURE 1:**
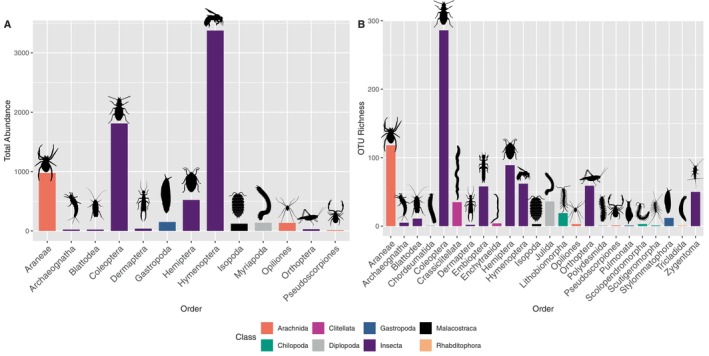
Abundance and OTU richness of arthropod orders across different taxonomic classes. (A) Total abundance of morphologically identified individuals (lowest feasible taxonomic level possible). (B) OTU richness by Order; taxonomic assignment was performed on 07/12/2023. Orders are categorized by their respective classes, indicated by color coding. Silhouettes extracted from PhyloPic (phylopic.org).

A total of 9418 individuals were counted and identified into 13 distinct taxonomic groups (mostly at the Order level) based on morphological identification (Quigley and Madge 1988; Figure 1A). Metabarcoding analysis identified 861 OTUs belonging to 24 different taxonomic orders. OTUs identified spanned across a diverse range of orders, including Araneae, Archaeognatha, Blattodea, Chordeumatida, Coleoptera, Crassiclitellata, Dermaptera, Embioptera, Enchytraeida, Hemiptera, Hymenoptera, Isopoda, Julida, Lithobiomorpha, Opiliones, Orthoptera, Polydesmida, Pseudoscorpiones, Pulmonata, Scolopendromorpha, Scutigeromorpha, Stylommatophora, Tricladida, and Zygentoma (Figure 1B). Interestingly, OTUs present in the metabarcoding samples were assigned to the manually removed Carabidae and Formicidae families.

**FIGURE 2:**
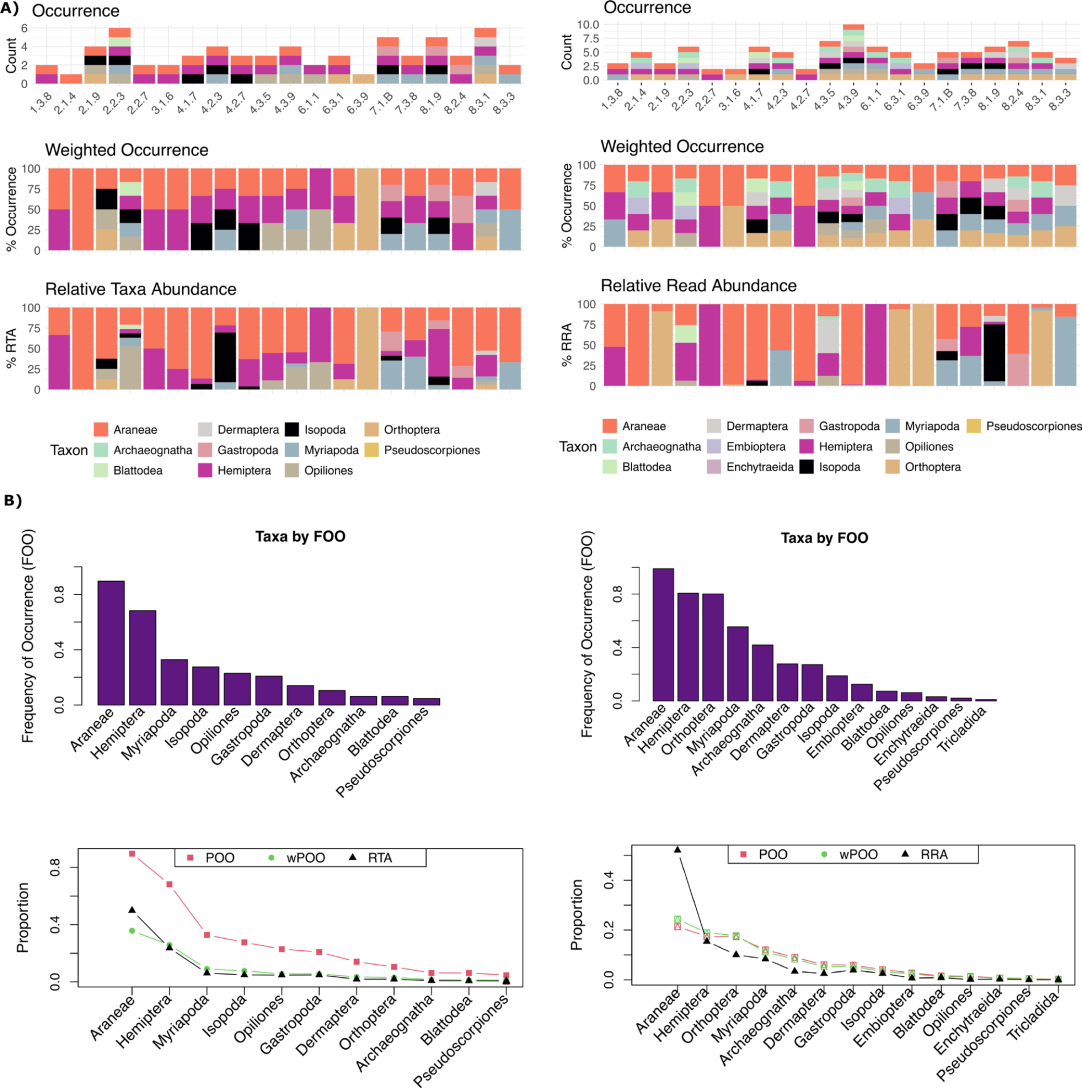
Comparison of biodiversity metrics between morphological identification and DNA metabarcoding at both the sample and population levels, adapted from Deagle et al. (2019). Data from the morphological analysis are presented on the left of each panel, while data from metabarcoding are on the right. (A) Community composition at the sample level for 20 representative samples. The top row shows the taxonomic richness (number of taxonomic groups detected per sample). The middle row shows the weighted occurrence of individuals (left) and the weighted read occurrence (right) for each taxon within each sample. The bottom row provides the relative taxa abundance, RTA (left) and the relative read abundance, RRA (right). (B) Community metrics at the population level across all samples. The top row shows the Frequency of Occurrence (FOO), the proportion of all samples in which a taxon was detected, for the 15 most frequent taxa in each dataset. The bottom row compares three summary metrics: POO (Percent of Occurrence): A presence/absence metric where the FOO of all taxa is rescaled to sum to 100%. In this metric, samples with higher taxonomic richness have a greater influence on the overall result. wPOO (Weighted Percent of Occurrence): Similar to POO, but each taxon's occurrence is weighted according to the number of taxa. RTA and RRA: The proportion of specimens or reads for a given taxon out of the total number of specimens or reads for all taxa, respectively.

The comparison between morphological and metabarcoding datasets revealed differences in both taxonomic richness and community composition (Figures 1 and 2A,B). Morphological identification showed higher total abundance in certain groups (e.g., Isopoda and Coleoptera), while metabarcoding captured substantially higher taxonomic richness at the order level, as reflected in OTU richness (Figure 1B). At the sample level (Figure 2A), both methods detected a broad range of taxa, but metabarcoding consistently revealed more taxa per sample, including those represented by low read counts. This is especially evident in the occurrence and weighted occurrence panels, where metabarcoding's sensitivity to rare OTUs leads to a more taxonomically diverse profile. However, when read abundance is taken into account (RRA, bottom right of Figure 2A), many of these taxa are shown to constitute only a small fraction of the overall sequence data, indicating low biological abundance. This contrasts with the Relative Taxa Abundance (RTA) in morphological data, where dominant taxa, such as Araneae, Myriapoda, and Isopoda, consistently account for the majority of individuals. At the community level (Figure 2B), Araneae emerged as the most frequently (FOO) detected order across both methods, followed by Myriapoda, Hemiptera, and Opiliones. The Frequency of Occurrence (FOO), Percent of Occurrence (POO), and Weighted Percent of Occurrence (wPOO) metrics illustrate how occurrence‐based summaries may inflate the importance of rare taxa, while abundance‐based summaries (RRA and RTA) more accurately reflect dominant community members.

Addressing the relationship between morphological abundance and the quantity of sequencing reads for the identified 13 taxonomic groups, mostly at the Order level (Appendix S1: Figure S5), a statistically significant correlation was identified for 12 of the 13 groups. This suggests that, overall, increased specimen abundance was associated with elevated read counts; however, its strength varied considerably. For instance, strong correlations were observed for orders such as Blattodea (Cor = 0.9, *p* value < 0.001) and Orthoptera (Cor = 0.86, *p* value < 0.001), but other taxa exhibited smaller, albeit substantial, positive correlations, including Opiliones (Cor = 0.43, *p* value < 0.001) and Isopoda (Cor = 0.33, *p* value < 0.001). The Pearson correlations for Coleoptera (Cor = 0.3, *p* value < 0.001) and Hymenoptera (Cor = 0.26, *p* value < 0.001) were notably low. Nonetheless, OTU reads associated with these families were still identified in the metabarcoding dataset. The only group with no significant correlation was Gastropoda (Cor = 0.05, *p* value = 0.46), where read counts did not reflect morphological abundance.

### 3.3 Evaluating Decontamination of Bulk Samples

**FIGURE 3:**
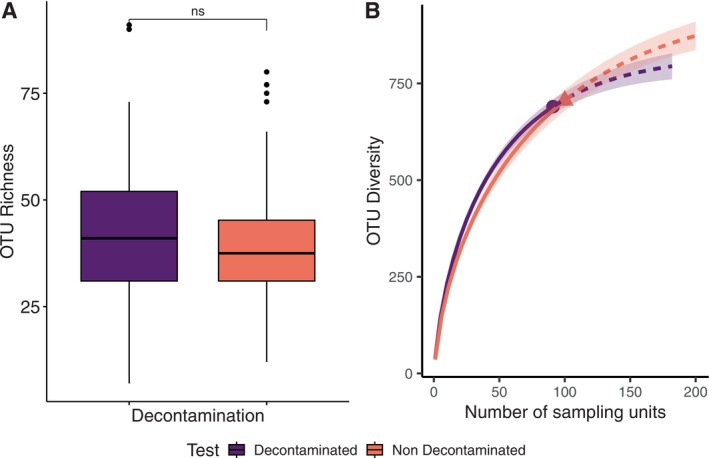
Comparison of OTU richness and diversity in decontaminated (purple) versus non‐decontaminated (orange) samples: (A) Boxplots comparing OTU richness between decontaminated and non‐decontaminated samples and analyzed using a two‐sample *t*‐test (ns, nonsignificant); (B) Rarefaction curves of OTU richness plot, based on an incidence matrix. Solid lines represent interpolation, dotted lines represent extrapolation, and shaded areas represent 95% confidence intervals.

The sodium hypochlorite (NaOCl) decontamination method did not show significant differences in OTU richness between decontaminated and non‐decontaminated samples (Figure 3A). Additionally, rarefaction curves for both sets of samples followed similar trends, indicating comparable levels of sequencing depth and species richness (Figure 3B).

### 3.4 Evaluation of OTU Recovery Efficiency

Morphologically, 660 carabid individuals were identified and classified into 31 species, the corresponding barcodes constituting the mock community. As expected, OTU richness varied with the addition of a mock sample (Appendix S1: Figure S7A). In high‐ and low‐diversity samples, OTU richness increased proportionally with the addition of mock community DNA regardless of the concentration added (Appendix S1: Figure S7A), suggesting a strong ability to recover taxonomic diversity through metabarcoding. However, in low‐diversity samples, richness estimates were less consistent and lower overall (Appendix S1: Figure S7A). The mock community generated 83 OTUs, of which 65 (77%) OTUs belonged to the Order Coleoptera, with 34 different OTUs belonging to the Carabidae family (Appendix S1: Figure S7B), consistent with the expected species composition. This consistency demonstrates the high resolution of metabarcoding for identifying dominant taxa within the sample.

### 3.5 Carabid Species‐Level Recovery Efficiency

**FIGURE 4:**
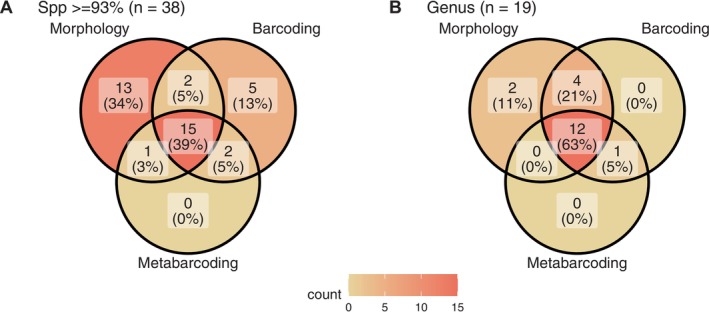
Comparison of identification methods at species and genus levels for carabid species using morphology, barcoding, and metabarcoding methods. (A) Species‐level analysis (*n* = 38) following the criteria established by Conrado et al. (2023), with a barcode gap threshold of ≤ 7% of homology (Appendix S1: Figure S6). A total of 38 species were identified, of which 18 were defined as Integrated Operational Taxonomic Units (IOTUs), seven as Molecular Operational Taxonomic Units (MOTUs) and 13 as species (Appendix S1: Table S2). Concordance among all three methods used was observed in 15 IOTUs (39%), while 34% of the species were only identified morphologically. (B) Genus‐level analysis (*n* = 19) of carabid taxa showed that 63% of the genera were identified by the three methods and taxonomy identifies 95% of the present genera. One carabid genus, *Acupalpus*, was exclusively detected by molecular methods.

ASAP analysis of the 671‐carabid COI sequence dataset revealed optimal species partitions with pairwise distance thresholds ranging from 0.031 to 0.078. The best‐supported partition corresponded to a threshold of 0.071 (i.e., 93% similarity), which was adopted as a conservative cutoff for tentative species‐level assignment. The comparative analysis of the three methods revealed a total of 38 unique carabid species identified at ≥ 93%. Indeed, most specimens from the mock community matched reference sequences with > 97% similarity. A single exception (*Poecilus cupreus*) showed 95.25% similarity, which we consider a borderline but acceptable match. Specimens with similarity values below 93% (e.g., *Carabus morbillosus*, 92.84%) were excluded from species‐level classification and not included in diversity metrics. These cases are flagged in the Supporting Information as “Not considered” (Appendix S1: Table S2). Morphological identification alone detected 31 species (Figure 4A). Single‐specimen barcoding generated 24 sequences, of which 17 are IOTUs and 7 are MOTUs, while metabarcoding of the mock community identified 18 sequences, 16 are IOTUs and two are MOTUs. The highest level of agreement, at the species level, was in the center of the diagram, with an overlap of 15 species (39%) detected by all three approaches: morphological identification, single‐specimen barcoding, and metabarcoding (Figure 4A). Each method showed discrepancies (Appendix S1: Table S2); morphology detected 13 species (34%) that were missed by both molecular methods. Single‐specimen barcoding identified five species (13%) that were missed by the other two methods, highlighting differences in detection capabilities between methods.

At the genus level, Figure 4B, the methods showed greater concordance, with 19 unique genera identified in total. A large majority of these, 12 genera (63%), were successfully identified by all three methods. Critically, addressing a key comparative question, we found one genus (5%) that was detected by both barcoding and metabarcoding but was absent from the morphological inventory.

## 4 Discussion

This study not only enriches the local reference database of cytochrome oxidase I (COI) mitochondrial gene sequences for carabid species in Portugal but also highlights the importance of integrating molecular techniques to enhance our understanding and conservation of soil ecosystems. Establishing accurate, reliable methods for soil fauna assessment is essential to fill knowledge gaps and improve biodiversity monitoring of soil communities and populations, which still rely heavily on existing taxonomic expertise.

### 4.1 The Value of Decontamination in DNA Metabarcoding Samples

Our findings highlight key considerations for optimizing biodiversity research methodologies and ensuring the reliability of future metabarcoding workflows. Prior research indicates that sodium hypochlorite bleaching does not compromise COI barcoding success, making it effective for decontaminating bulk DNA samples by removing external contaminants and reducing cross‐sample contamination (Hausmann et al. 2021; Jüds et al. 2023). This procedure can be time‐consuming and, as our results indicate, there is no significant difference in OTU richness between decontaminated and non‐decontaminated samples (Figure 3A), as further evidenced by similar rarefaction curves (Figure 3B). This suggests that decontamination did not alter the overall taxonomic composition and, therefore, may not be essential for ensuring data reliability when appropriate sampling designs and careful sample handling are in place. While decontamination can be valuable (Hausmann et al. 2021), it may not always be essential, especially when robust sampling and handling protocols are in place. Researchers should consider decontamination based on specific research objectives, available resources, and sample‐handling practices. Additionally, it is essential to recognize the limitations of decontamination; its effectiveness can vary with target taxa, environmental conditions, and specific protocols, underscoring the need for careful evaluation in molecular ecology studies. To further control contamination sources, incorporating negative controls throughout the sampling process is recommended (Liu et al. 2020).

### 4.2 The Importance of Using Mock Communities

For each sample where the mock community was added, regardless of concentration, the OTU richness increased as expected (Appendix S1: Figure S7A). This result contributes to validating metabarcoding as a reliable method to recover OTUs regardless of their input proportions. The mock community consisting of 31 barcoded species of Carabidae identified morphologically, provided a robust benchmark for assessing the efficiency of metabarcoding pipelines. The observed OTU richness and taxonomic composition were expected; 83 OTUs were identified in the mock community sample, particularly for Carabidae (Appendix S1: Figure S7B). Taxa belonging to other coleoptera families or the other orders present may be due to secondary amplification, such as gut content. This suggests that metabarcoding is an effective tool for recovering dominant taxa.

Metabarcoding of the mock community sample assigned 34 OTUs to the Carabidae family (Appendix S1: Figure S7B), slightly exceeding the expected number of 31 species. The overestimation observed in assigning OTUs can be attributed to a confluence of factors: the complexities of intraspecific variation, the unpredictable nature of sequencing artifacts, the nuances of secondary amplification, and the biases that may emerge from bioinformatic pipelines (Keck et al. 2023). Using a mock community of Carabidae to validate our metabarcoding pipeline demonstrated that while both barcoding and metabarcoding techniques identified carabid species, certain inconsistencies arose, underscoring the value of integrating multiple methods for accurate species identification (Janzen et al. 2005; Salis et al. 2024). Moreover, while standard species‐level thresholds for COI barcoding often exceed 97%, we adopted a 93% similarity cutoff based on an ASAP analysis tailored to our 231 bp fragment. This empirically derived threshold balanced resolution with caution, especially given the limited length and variability of the marker. Most specimens exceeded 97% similarity, with only one borderline case (*Poecilus cupreus*, 95.25%) retained based on consistent morphological and metabarcoding evidence. Lower similarity matches were excluded from species‐level interpretation to avoid overclassification. In practice, all but one mock community specimen exceeded 97% similarity to reference sequences, supporting the appropriateness of the approach, and consistent with established barcode gap values for carabids (Pentinsaari et al. 2014; Raupach et al. 2016). Also, in this context, the barcode gap was not used here to delimit species in a strict taxonomic or phylogenetic sense but rather as a practical threshold to define a similarity threshold for species‐level retention within the bioinformatic pipeline, an approach that facilitates consistency and comparability across metabarcoding studies.

Due to the filtering of OTUs according to the barcode gap (Appendix S1: Figure S6), of the 34 OTUs, only 18 remained for the methodological comparison. This reinforces that the effectiveness of DNA metabarcoding relies on the quality, comprehensiveness and accuracy of DNA sequence databases, as taxonomic sequence assignment requires matching unknown DNA sequences against reference databases (Keck et al. 2023; Taberlet et al. 2018). Our findings highlight the importance of integrative approaches to address the limitations of each method, allowing for more reliable species assignments (Conrado et al. 2023; Cristescu 2014; Janzen et al. 2005; Salis et al. 2024).

### 4.3 The Use of Integrative Approaches

Collecting biodiversity data on soil macrofauna remains challenging due to the lack of standardized sampling procedures and difficulties in taxonomic assignment (Cameron et al. 2018; Guerra et al. 2022). In our study, sampling across the 24 sites yielded a substantial dataset comprising 9418 individuals representing a diverse array of taxa.

Morphological classification sorted these specimens into 13 taxonomic groups (mostly orders) (Figure 1A), providing a baseline understanding of the macroscopic biodiversity within the study area. Importantly, our metabarcoding approach significantly enhanced taxonomic resolution, increasing to 24 orders as a result of 861 OTUs identified. As seen in other recent studies, molecular techniques often reveal more diversity at the same taxa level than morphological approaches alone (Arjona et al. 2022; Mata et al. 2021), making metabarcoding an invaluable tool for comprehensive and practical assessments. A balanced comparison requires acknowledging the inherent limitations of the morphological dataset used as our benchmark. Morphology relies on expressed phenotypic characters, which may be absent or ambiguous, whereas DNA metabarcoding interrogates genetic information directly. Metabarcoding not only allowed a finer‐scale taxonomic resolution but also uncovers cryptic diversity that might have been overlooked through traditional morphological methods alone, such as damaged specimens, larval stages and cryptic species complexes (Salis et al. 2024), that have been found in different soil invertebrates, for example in beetles (Mitchell et al. 2020; Pérez‐Delgado et al. 2022) earthworms (King et al. 2008) and pseudoscorpiones (Pérez‐Delgado et al. 2022). Overall, our comparison of morphological and metabarcoding data revealed clear differences in both taxonomic richness and community composition. While morphological identification provided robust estimates of dominant taxa such as Isopoda and Coleoptera, metabarcoding recovered substantially higher taxonomic richness, particularly by detecting cryptic or low‐abundance taxa (Figure 1). At the sample level, metabarcoding consistently identified more taxa per sample, especially through occurrence‐based metrics (Figure 2A). However, when relative abundance was considered, many of these taxa contributed only minimally to the total read counts, highlighting the potential overrepresentation of rare taxa in presence/absence‐based summaries. At the community level (Figure 2B), metrics such as Frequency of Occurrence (FOO), Percent of Occurrence (POO), and Weighted POO (wPOO) emphasized widespread taxa like Araneae and Hemiptera but also showed how different metrics can shift the perceived importance of rare versus dominant taxa. Abundance‐based metrics (RRA/RTA) provided a more conservative reflection of community structure, consistent with the expected biological dominance. Importantly, this study represents, to our knowledge, the first application of biodiversity metrics commonly used in dietary metabarcoding, originally described by Deagle et al. (2019), to non‐dietary community data. Our results demonstrate that these metrics, when applied beyond diet studies, offer valuable perspectives on community structure and taxonomic detection patterns. As in dietary applications, occurrence‐based metrics are useful but can exaggerate the role of rare taxa, whereas abundance‐based metrics provide stronger inferences about ecological dominance. Together, these findings highlight the complementary value of morphological and molecular data and show how adopting diverse summary metrics can enhance our understanding of biodiversity patterns in soil macrofaunal communities.

Taxonomic resolution plays a central role in biodiversity assessments, and we emphasize the importance of transparency when reporting identifications, particularly, those retained at higher taxonomic levels in both classic and molecular‐based approaches (Keck et al. 2023). In our study, several taxa were conservatively classified at the Family or Order level due to insufficient morphological resolution or limited taxonomic expertise, which prevented us from confidently assigning specimens to genus or species. While such higher level assignments reduce specificity, they enhance reliability and reproducibility. For molecular identification, we utilized the curated BOLD reference database, which enables species‐level resolution when reference sequences are available. However, we acknowledge the limitations of this approach, such as incomplete database coverage, regional gaps, reduced marker resolution, and taxonomic ambiguities, which may restrict accurate assignments to higher taxonomic ranks (Keck et al. 2023; Sheth and Thaker 2017). These factors call for cautious interpretation and highlight the need for ongoing investment in reference library development and taxonomic expertise. Therefore, discrepancies in which metabarcoding detects taxa missed by morphology do not necessarily reflect error in visual identification but rather illustrate the molecular method's capacity to access taxonomic resolution beyond the limits of morphological analysis. Moreover, metabarcoding can detect DNA from organisms not physically present in the final sample. For example, in our study, we detected OTUs assigned to Formicidae and Carabidae despite the deliberate removal of these specimens prior to homogenization. This commonly observed phenomenon in bulk sample analysis is likely due to the amplification of “secondary DNA,” such as predator gut content DNA or DNA transfer between specimens via preservative fluid (Pompanon et al. 2012). These findings underscore a defining feature of metabarcoding: it inventories all DNA present in a sample and not just from intact, observable organisms. While this enhances detection sensitivity, it also necessitates careful ecological interpretation, especially when results diverge between molecular and morphological methods.

The enhanced resolution, combined with the ability to detect residual DNA underscores the value of metabarcoding as a tool combined with traditional taxonomy and barcoding, enabling more thorough and accurate biodiversity assessments (Conrado et al. 2023; Cristescu 2014; Janzen et al. 2005; Salis et al. 2024) and revealing hidden layers of soil biodiversity essential for conservation and ecosystem management. While our findings show that molecular methods can improve taxonomic assignment and help detect taxa not recovered through morphology alone, we did not directly assess genetic diversity or cryptic species.

A challenge in metabarcoding is determining whether sequence read counts can serve as a reliable proxy for species relative proportions (Deagle et al. 2019). Our results demonstrate that while a positive relationship often exists, it is highly dependent on, and variable across taxa (Appendix S1: Figure S5). The wide variation in correlation strengths across orders likely stems from a combination of biological and technical biases, including taxa differences in biomass, DNA extraction efficiency, and PCR primer affinity. The weakened correlations in Coleoptera and Hymenoptera provide a powerful, practical demonstration of this. The removal of the highly abundant Carabidae and Formicidae families from the molecular workflow disrupted the correlation between the total morphological count and the resulting sequence data. This highlights that metabarcoding read counts reflect the composition of the processed sample, not necessarily the original community. Experimental decisions such as sample sorting can have impacts on quantitative interpretations. Furthermore, the nonsignificant correlation in Gastropoda could be attributed to poor primer‐template affinity for molluscan DNA, inefficient DNA extraction or a low number of individuals. Ultimately, our findings support the view that while read counts are not a direct measure of specimen abundance, they can provide valuable insights when interpreted with a clear understanding of the methodological biases inherent to the specific workflow and focal taxa.

### 4.4 The Importance of Comprehensive and Reliable Reference Databases

Molecular reference database platforms, such as Bold Systems and NCBI, provide valuable resources for DNA metabarcoding. However, species‐level assignment reliability remains a topic of discussion (Leray et al. 2020; Locatelli et al. 2020). Challenges are particularly pronounced for less‐researched invertebrate taxa (Leray et al. 2020), whereas assignments at the genus or higher taxonomic levels are generally more reliable (Leray et al. 2019). Our results reflect this trend. As shown in Figure 4, the strong intersection at the genus level (63%) implies a higher concordance between methods for genus identification compared to species (39,%); accordingly a higher rate of assignment discrepancies was found at the species level compared to the genus level (Figure 4).

These discrepancies may be caused by mismatches between global reference sequences and the local species compositions of the studied ecosystem, which can impair molecular identifications, as a sequence can only be reliably assigned to a species if its sequence has been previously vouchered (Arjona et al. 2022). Vouchered species should contain information regarding their DNA barcode, key morphological traits, and a bibliography for identification (Cao et al. 2016; Collins and Cruickshank 2013). Therefore, biodiversity research should not rely exclusively on reference databases for molecular identification of species, and these databases must be continuously updated and curated (Keck et al. 2023). In our study, the barcoding assessment of Carabidae revealed cases where sequences did not match any known references, likely due to genetic divergence or underrepresentation in existing databases (Arjona et al. 2022; Keck et al. 2023). The development of high‐quality reference databases, particularly local barcode reference libraries based on established taxonomic knowledge, is essential to increase the reliability of molecular approaches (Cristescu 2014; Janzen et al. 2005; Keck et al. 2023; Salis et al. 2024). For example, updating the BOLD database with our carabid voucher sequences is expected to reduce the proportion of species identified solely through morphological methods and increase congruence across methods. This is projected to shift our Venn diagram results (Figure 4A) from the current 34% of species identified only by taxonomy to nearly zero while boosting the overlap across methods.

## 5 Conclusions and Recommendations

This study highlights the advancements of molecular approaches for soil macrofauna biodiversity assessments, particularly DNA barcoding and metabarcoding. We can overcome current limitations by fostering integrative research of both molecular and morphological taxonomic methodologies and developing robust reference libraries, improving data accuracy, and deepening our understanding of complex ecosystems. This incorporation offers significant potential for advancing global and regional biodiversity research and ecological monitoring as researchers can bridge taxonomic gaps, increase resolution, and achieve a deeper understanding of local biodiversity. However, it is critical to establish robust local barcode references.

Our findings support that while morphological classification provides a solid baseline, molecular methods complement it by improving taxonomic assignment in some cases, detecting taxa that were overlooked morphologically, and contributing to a more complete picture of the sampled carabid community. Although our data did not specifically uncover cryptic species or assess genetic diversity patterns in detail, the combined approach strengthens biodiversity assessments and holds promise for future ecological monitoring. Additionally, we emphasize the need to continuously update, review and expand global databases, ensuring the accuracy and reliability of molecular identifications, as one of the major challenges in molecular biodiversity research lies in the limitations of reference databases. Curating species‐level annotations will require a substantial scientific effort and close interdisciplinary collaboration, involving molecular ecologists and experts from each taxonomic group. For future research, we recommend:

1. Enhancing Local Reference Libraries: Invest in developing and curating local barcode references that capture species' genetic diversity at regional or even local scales. This step is essential for accurate species assignments, particularly for soil invertebrates with high cryptic diversity.
2. Integrative Taxonomic Approaches: Employ both morphological and molecular methods to maximize taxonomic resolution, enabling researchers to bridge gaps in species identification and reveal cryptic taxa.
3. Refining Decontamination Protocols: Investigate the effects of decontamination across different taxa, environmental contexts, and sample types to optimize protocols for molecular studies. Employing negative controls throughout the sampling and laboratory components is also advised to control contamination sources.
4. Standardizing the Use of Negative Controls: Consistently incorporate negative controls throughout field sampling and laboratory workflows to detect and mitigate potential contamination, ensuring the reliability of molecular data.
5. Evaluating Quantitative Relationships: Future studies should systematically investigate how specimen abundance, biomass, and population density relate to sequencing read counts across taxa. Understanding these relationships is critical for assessing the quantitative reliability of metabarcoding and enhancing its application in ecological monitoring and population‐level analyses.
6. Database Development and Maintenance: Prioritize the systematic updating of reference databases with voucher‐based sequences to enhance the reliability of molecular identifications.
7. Focus on relationships between demographic parameters such as biomass and density with read counts might provide insights into population dynamics

Ultimately, combining molecular and traditional taxonomic methods offers a promising way to address current limitations by fostering integrative research methodologies, developing robust reference libraries, and improving data accuracy. This approach has the potential to enhance biodiversity research, deepen our understanding of species interactions and environmental health, improve existing ecosystem management practices, and optimize conservation strategies.

## Author Contributions

**Luísa Fraga Dornellas:** data curation (lead), formal analysis (lead), investigation (equal), project administration (equal), resources (equal), software (lead), validation (equal), visualization (equal), writing – original draft (lead), writing – review and editing (equal). **Vanessa A. Mata:** data curation (supporting), formal analysis (supporting), investigation (supporting), methodology (supporting), resources (supporting), software (supporting), supervision (supporting), writing – review and editing (equal). **Sara Mendes:** formal analysis (supporting). **Ricardo Leitão:** investigation (supporting), methodology (supporting), writing – review and editing (supporting). **Marie Bartz:** investigation (equal), methodology (supporting). **Eduardo Nascimento:** investigation (supporting). **Joana Costa:** conceptualization (equal), funding acquisition (equal), methodology (equal), project administration (equal). **José Paulo Sousa:** funding acquisition (equal), methodology (equal), project administration (equal), supervision (equal). **Luís Cunha:** conceptualization (lead), data curation (equal), formal analysis (equal), funding acquisition (equal), investigation (equal), methodology (lead), project administration (equal), supervision (lead), validation (supporting), writing – review and editing (lead).

## Conflicts of Interest

The authors declare no conflicts of interest.

## Supporting information


**Appendix S1:** ece372461‐sup‐0001‐AppendixS1.docx.

**Appendix S2:** ece372461‐sup‐0002‐AppendixS2.xlsx.

## Data Availability

All data, code, and bioinformatic pipelines used in this study are available on Dryad through the link https://doi.org/10.5061/dryad.g1jwstr28. All carabid sequence GenBank accession numbers are also available in Appendix S2.
