# Supplementary material for: Establishing DNA‐Based Strategies for Soil Biodiversity Assessment: Insights From Carabid Beetles
Source: Ecol Evol. 2025 Nov 6;15(11):e72461. doi: 10.1002/ece3.72461 (PMC12592676; doi:10.1002/ece3.72461)
Supplement: Supplementary file 1 — Appendix S1: ece372461‐sup‐0001‐AppendixS1.docx. [file ECE3-15-e72461-s002.docx]

Supplementary Materials A


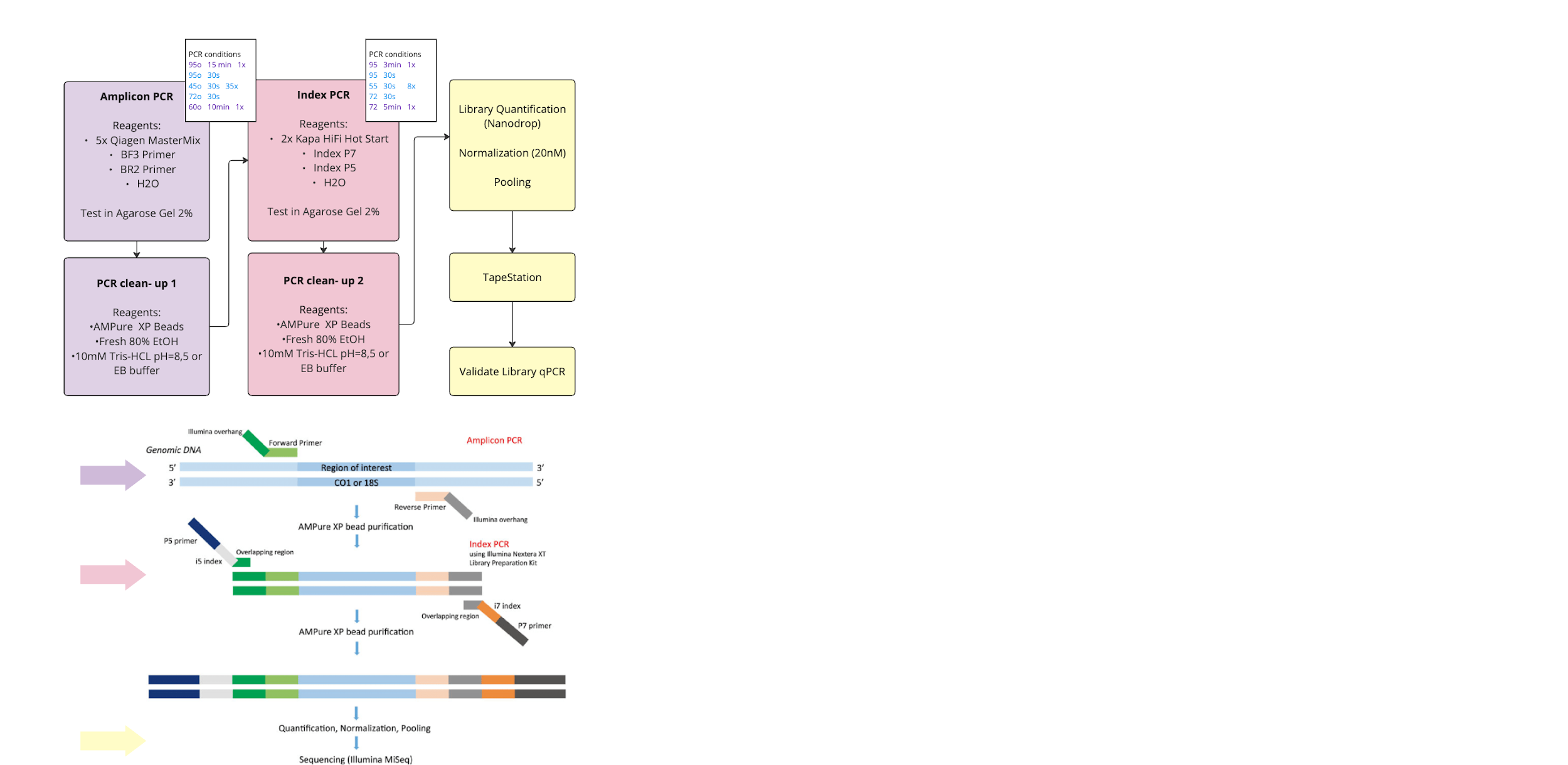


Fig. 1: Dual-PCR procedure for Illumina library preparation (Amplicon PCR + Index PCR). The first PCR step, in purple, uses amplicon-specific primers (BF3 and BR2) including 7bp Illumina adapter overhangs. Second PCR allows the incorporation of Illumina index adapters i5 and i7. Following each process, bead purification is performed. Prior to sequencing on the Illumina Novaseq, quantification, normalization, and pooling are performed. Adapted from Bourlat et al. (2016) and Illumina (2013).


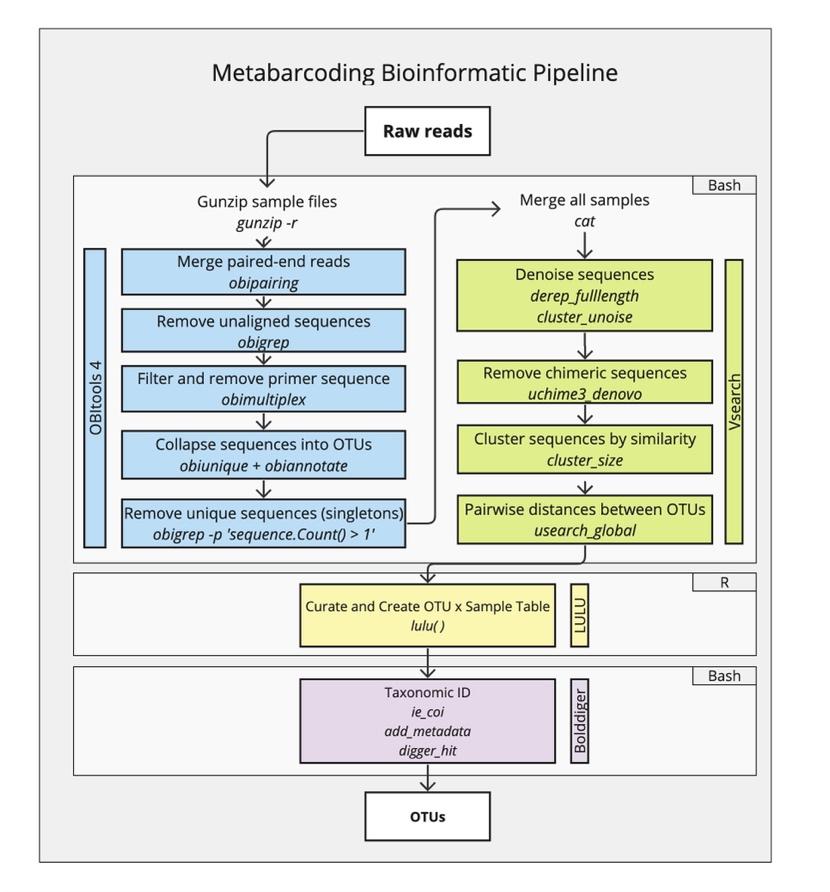


Fig. 2: Flowchart illustrating the steps involved in the metabarcoding bioinformatic pipeline. Raw reads are processed through various stages including sample file merging, sequence alignment and filtering, primer sequence removal, OTU clustering, denoising and chimaera removal steps, with the final output being curated OTUs annotated with taxonomic IDs. The processes are carried out using a combination of Bash and R scripts using the packages Obitools 4 (Boyer et al., 2016), Vsearch (Rognes et al., 2016), LULU (Frøslev et al., 2017) and Boldigger (Buchner & Leese, 2020).

Table 1. Summary of data processing and filtering steps in the bioinformatics pipeline. "Count" refers to the number of sequences or OTUs remaining after the step, while "Removed" indicates the number discarded at that stage of the pipeline.


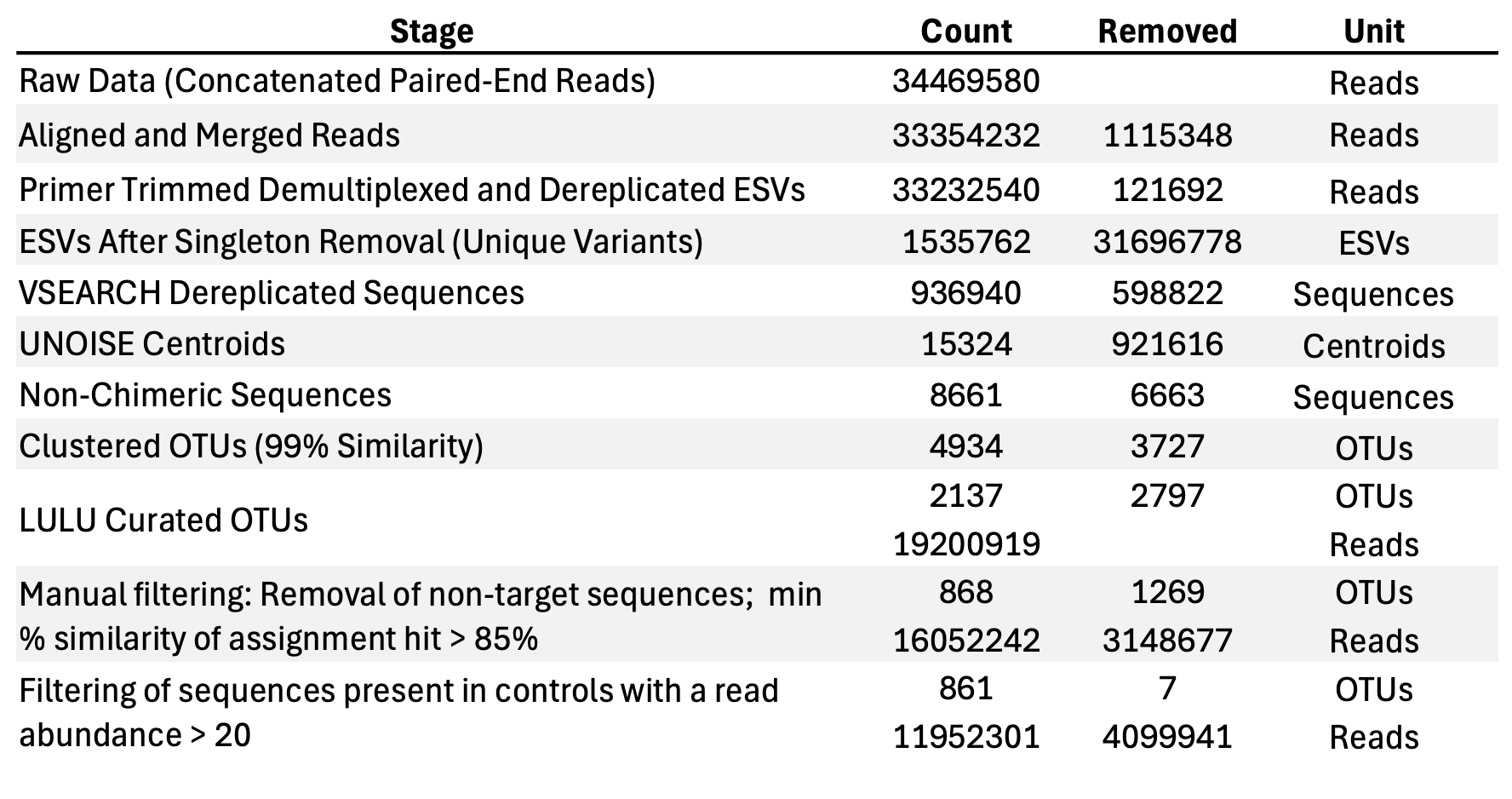


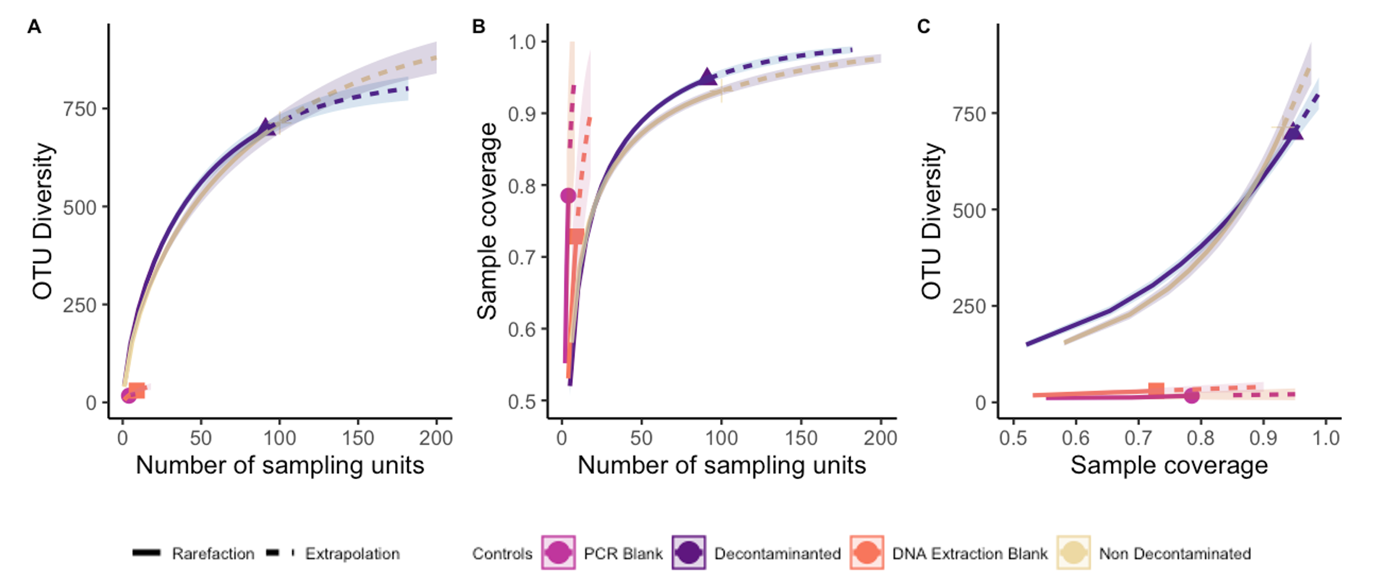


Fig. 3. Plots of Hill number q = 0 (species richness) of the richness of OTUs identified groups by sampling unit based on an incidence matrix of different test samples, PCR blanks (n = 4), samples decontaminated with sodium hypochlorite (n = 91), and DNA extraction blank controls (n = 9) samples non decontaminated (n = 100). Solid lines represent interpolation, while dotted lines represent extrapolation. (A) Sample-size-based curves; (B) sample completeness curve: accumulation of species with increasing sampling effort; (C) coverage-based curves: expected diversity as a function of expected coverage.


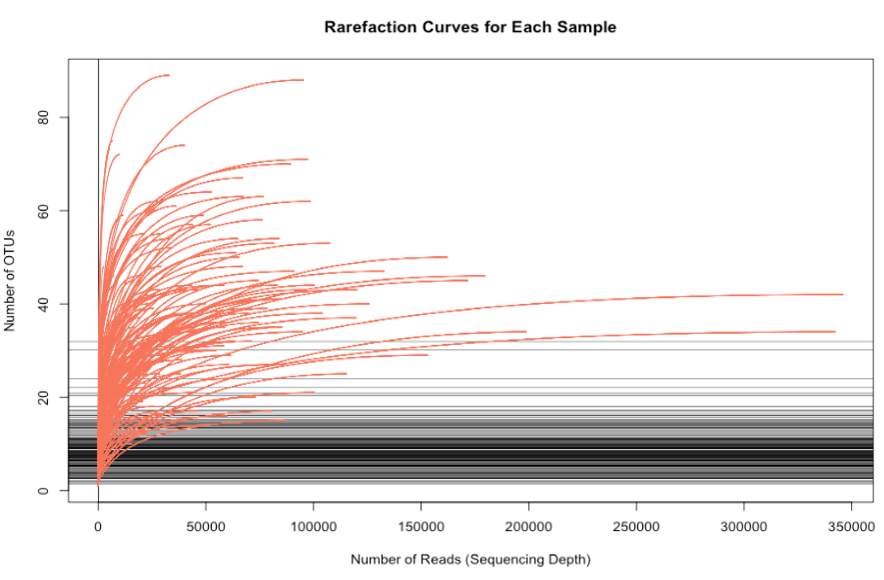


Fig. 4. Rarefaction curves of OTU richness against sequencing depth for each sample. Each curve represents a single sample from the DNA metabarcoding analysis. The x-axis shows the number of reads sampled (sequencing depth), and the y-axis shows the corresponding number of unique Operational Taxonomic Units (OTUs) detected. The asymptotic nature of the majority of the curves indicates that the sequencing depth was sufficient to capture most of the taxonomic diversity present within each individual sample library.


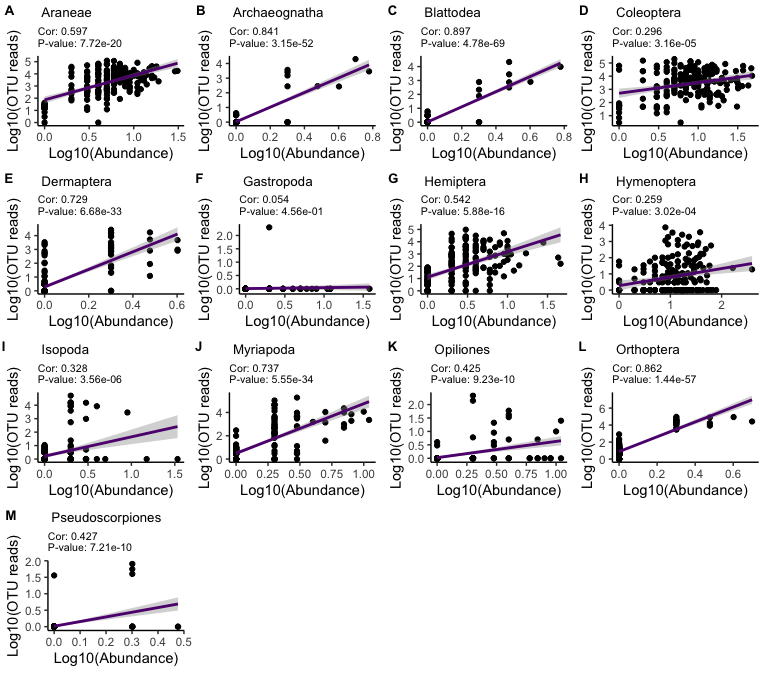


Fig. 5. Correlation between morphological abundance and DNA metabarcoding read counts. Scatter plots display the log10-transformed morphological abundance (x-axis) against log10-transformed number of OTU reads (y-axis) for 13 taxonomic groups: (A) Araneae, (B) Archaeognatha, (C) Blattodea, (D) Coleoptera, (E) Dermaptera, (F) Gastropoda, (G) Hemiptera, (H) Hymenoptera, (I) Isopoda, (J) Myriapoda, (K) Opiliones, (L) Orthoptera, and (M) Pseudoscorpiones. Each point represents a single sample. Purple lines represent the linear regression fit with the shaded area representing the 95% confidence interval. Pearson correlation coefficients (Cor) and p-values are indicated for each plot. Statistically significant positive correlations (p < 0.05) suggest a strong relationship between abundance and number of reads in these taxa, with the exception of Gastropoda (E), where the correlation is not significant. Note: Individuals from the Carabidae and Formicidae families were removed from the samples prior to DNA metabarcoding. The morphological abundance of the orders Coleoptera (D) and Hymenoptera (H) includes these families, but the read counts do not.


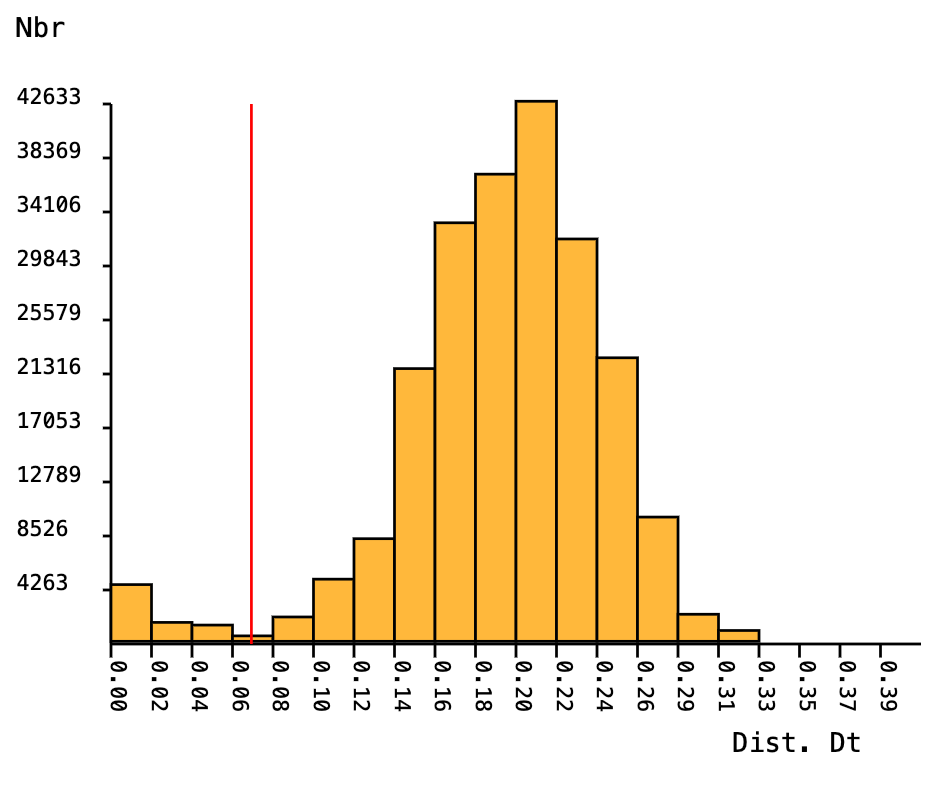


Fig. 6. Barcode Gap Histogram for Carabid Sequences. Histogram depicting the molecular distance (Kimura-2 parameter) versus frequency for Carabid sequences from a local reference barcode database and NCBI submissions (retrieved on 13/03/2024, NCBI sequence IDs available in Supplementary Materials B). The data is based on the cytochrome oxidase I (COI) gene and analyzed using Assemble Species by Automatic Partitioning (ASAP) species delimitation (Puillandre et al., 2021). The red line at y = 0.07 indicates the threshold distance (0.071) with the best score, highlighting the barcode gap.


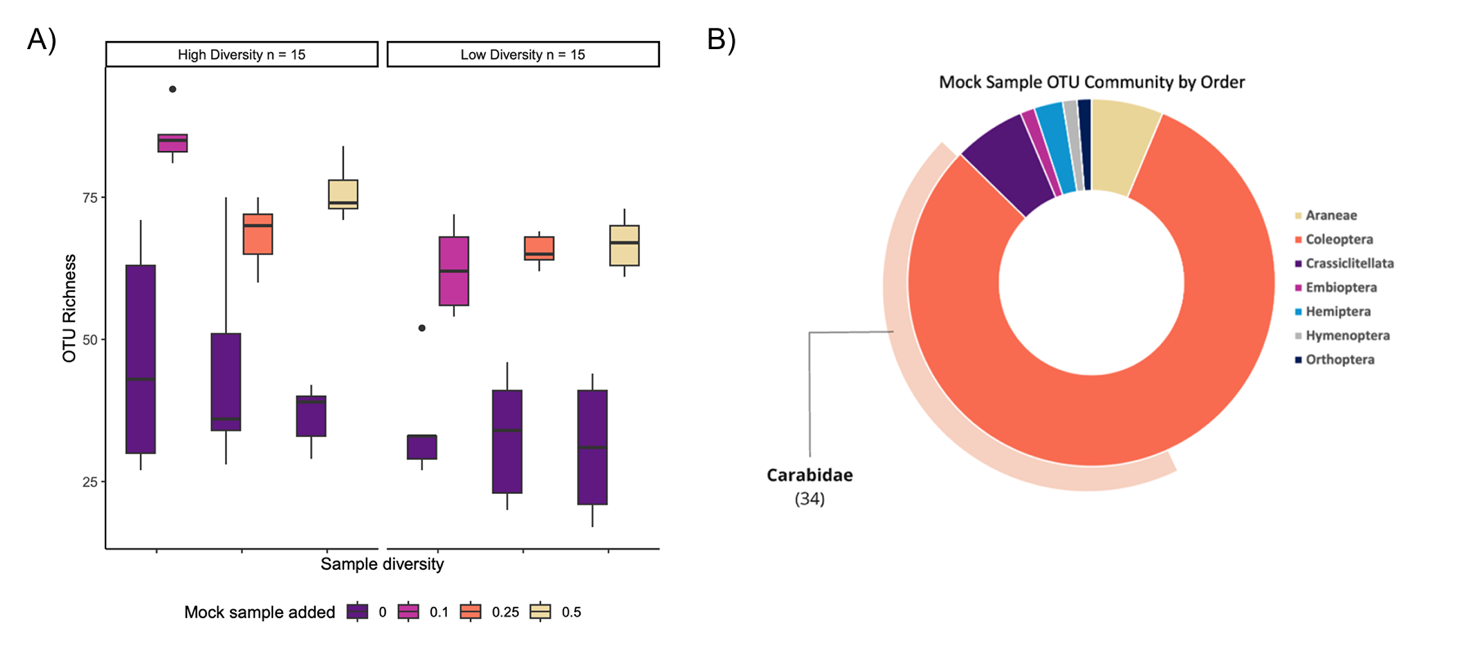


Fig. 7. (A) Boxplots of OTU richness trends for high- (n = 15) and low-diversity (n = 15) samples with varying proportions of mock community DNA added: 0 (n = 5), 0.1 (n = 5); 0.25 (n = 5) and 0.50 (n = 5). (B) Donut Plot displaying the OTU taxonomic composition of the mock community (n = 83) according to their Order, highlighting the dominance of Coleoptera and Carabidae. In total 8 Orders were present, where Coleoptera corresponded to 77% of the OTUs (65 OTUs), from which 34 OTUs belonged to the Carabidae family.

Table 2. Classification of carabids identified by each method, taxonomy, barcoding and metabarcoding, according to Conrado et al. (2023) Integrated Operational Taxonomic Units (IOTUs) and Molecular Operational Taxonomic Units (MOTUs) delimitation. IOTUs classified species morphologically identified with molecular data available; MOTUs classified species with only molecular data available. In taxonomy, “1” represents an identified morphospecies by the taxonomist and “0” species that were not identified. In the molecular methods, barcoding and metabarcoding the “0” represent species not identified by the method; only molecular data within a genetic similarity of >93% (0–7 % of homology) were considered for species classification.
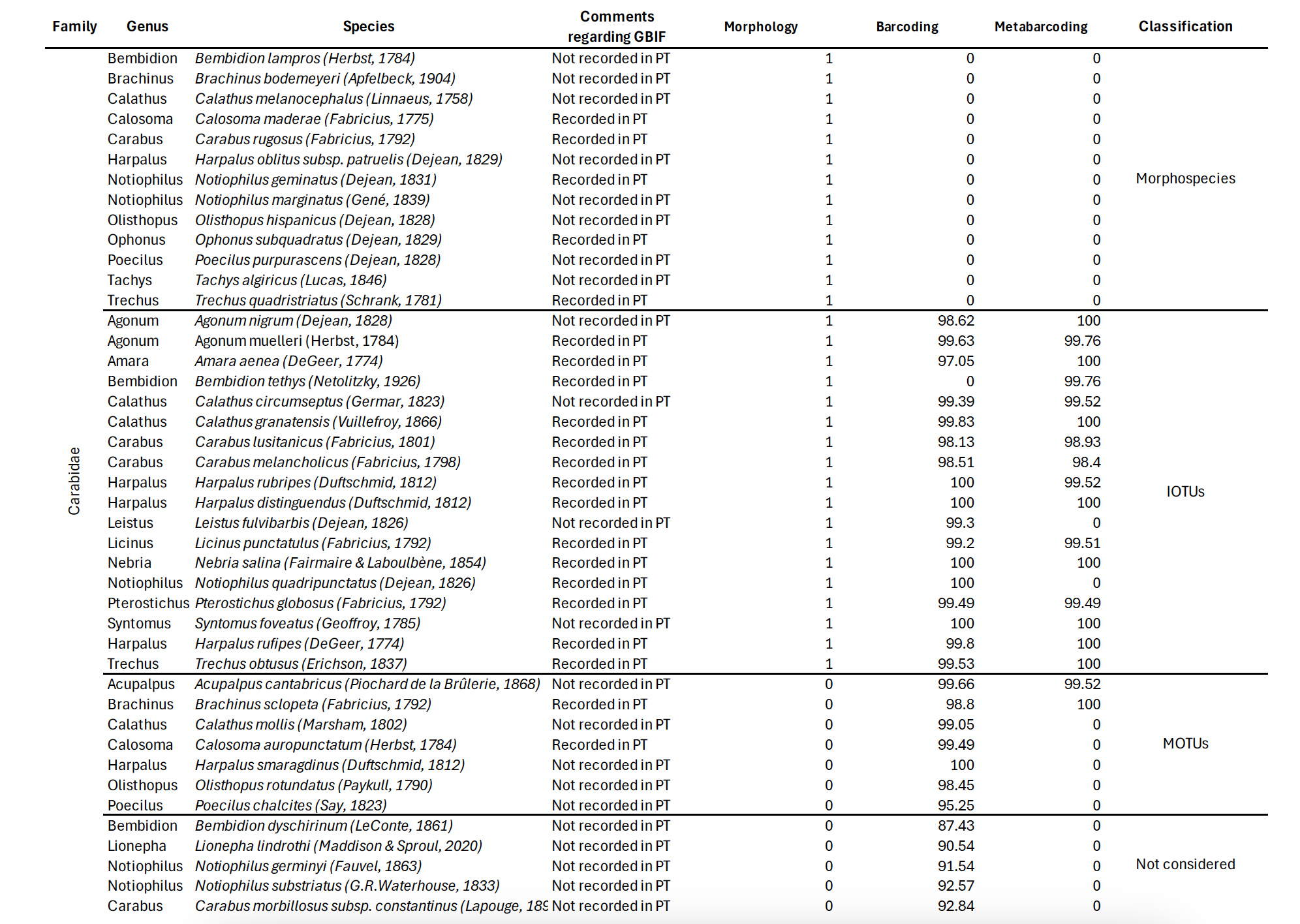
Comments correspond to the existence or not of records of the species in Portugal (PT).

**References**

Bourlat, S. J., Haenel, Q., Finnman, J., & Leray, M. (2016). Preparation of Amplicon Libraries for Metabarcoding of Marine Eukaryotes Using Illumina MiSeq: The Dual-PCR Method. *Methods in Molecular Biology (Clifton, N.J.)*, *1452*, 197–207. https://doi.org/10.1007/978-1-4939-3774-5_13

Boyer, F., Mercier, C., Bonin, A., Le Bras, Y., Taberlet, P., & Coissac, E. (2016). obitools: A unix-inspired software package for DNA metabarcoding. *Molecular Ecology Resources*, *16*(1), 176–182. https://doi.org/10.1111/1755-0998.12428

Buchner, D., & Leese, F. (2020). BOLDigger – a Python package to identify and organise sequences with the Barcode of Life Data systems. *Metabarcoding and Metagenomics*, *4*, e53535. https://doi.org/10.3897/mbmg.4.53535

Conrado, A. C., Demetrio, W. C., Stanton, D. W. G., Bartz, M. L. C., James, S. W., Santos, A., da Silva, E., Ferreira, T., Acioli, A. N. S., Ferreira, A. C., Maia, L. S., Silva, T. A. C., Lavelle, P., Velasquez, E., Tapia-Coral, S. C., Muniz, A. W., Segalla, R. F., Decaëns, T., Nadolny, H. S., … Cunha, L. (2023). Amazonian earthworm biodiversity is heavily impacted by ancient and recent human disturbance. *Science of The Total Environment*, *895*, 165087. https://doi.org/10.1016/j.scitotenv.2023.165087

Frøslev, T. G., Kjøller, R., Bruun, H. H., Ejrnæs, R., Brunbjerg, A. K., Pietroni, C., & Hansen, A. J. (2017). Algorithm for post-clustering curation of DNA amplicon data yields reliable biodiversity estimates. *Nature Communications*, *8*(1), Article 1. https://doi.org/10.1038/s41467-017-01312-x

Illumina. (2013). *16S metagenomic sequencing library preparation guide*. https://support.illumina.com/documents/documentation/chemistry_documentation/16s/16s-metagenomic-library-prep-guide-15044223-b.pdf

Puillandre, N., Brouillet, S., & Achaz, G. (2021). ASAP: Assemble species by automatic partitioning. *Molecular Ecology Resources*, *21*(2), 609–620. https://doi.org/10.1111/1755-0998.13281

Rognes, T., Flouri, T., Nichols, B., Quince, C., & Mahé, F. (2016). VSEARCH: A versatile open source tool for metagenomics. *PeerJ*, *4*, e2584. https://doi.org/10.7717/peerj.2584
